# Editorial: From Structure to Function in Neuronal Networks: Effects of Adaptation, Time-Delays, and Noise

**DOI:** 10.3389/fnsys.2022.871165

**Published:** 2022-04-21

**Authors:** Joana Cabral, Viktor Jirsa, Oleksandr V. Popovych, Alessandro Torcini, Serhiy Yanchuk

**Affiliations:** ^1^Life and Health Sciences Research Institute, School of Medicine, University of Minho, Braga, Portugal; ^2^Centre for Eudaimonia and Human Flourishing, Linacre College, University of Oxford, Oxford, United Kingdom; ^3^Center for Music in the Brain, Department of Clinical Medicine, Aarhus University, Aarhus, Denmark; ^4^Aix Marseille University, INSERM, INS, Institut de Neurosciences des Systèmes, Marseille, France; ^5^Institute of Neuroscience and Medicine, Brain and Behaviour (INM7), Research Centre Jülich, Jülich, Germany; ^6^CY Cergy Paris Université, Laboratoire de Physique Théorique et Modélisation, CNRS, UMR 8089, Cergy, France; ^7^Research Department Complexity Science, Potsdam Institute for Climate Impact Research, Potsdam, Germany; ^8^Department of Mathematics, Faculty of Mathematics and Natural Sciences, Humboldt-Universität zu Berlin, Berlin, Germany

**Keywords:** neuronal networks, adaptation, structure and function, noise, time-delay, neuronal activity patterns, multi-scale

It is a fundamental challenge to understand how brain function is related to its functional and structural organization, i.e., what shapes the neuronal activity patterns observed across scales that define cognitive and behavioral processes, as well as their breakdown in mental health disorders (Park and Friston, 2013). Few theories integrate the various dimensions in this ambitious endeavor [such as Free Energy Principle (Friston et al., 2006) and Structured Flows on Manifolds (Jirsa and Sheheitli, 2022)], but all acknowledge the multi-scale organization of brain function. Investigation of the complex structure-function relationship can be performed at the macro- and meso-scopic levels (Messé, 2020; Suárez et al., 2020), where dynamical modeling at large scales constitutes one of the promising methodologies (Ghosh et al., 2008; Deco et al., 2009; Honey et al., 2009). At the microscopic level, the dynamics of neuronal networks strongly depends on intrinsic properties of the neuro-anatomical connectome and the functional relationships among neurons, and this goes beyond the connectivity matrix. In particular, the adaptation of the strengths of the synaptic connections through synaptic plasticity (Markram et al., 1997; Abbott and Nelson, 2000; Dan and Poo, 2004), the evolution of the functional connectivity in time, the inevitable time delays resulting from both neurophysiological time constants and finite propagation velocity, noise, and inherent inhomogeneities play key roles in the emergent behavior of neuronal systems across spatial and temporal scales (Deco et al., 2009). A detailed characterization of these effects on the collective dynamics of neuronal networks is an important contemporary problem, which may thus provide the means for studying the link between functional and structural connectivity and brain function in health and disease (Cabral et al., 2017; Jirsa et al., 2017; McIntosh and Jirsa, 2019; Popovych et al., 2019).

This Research Topic focuses on the structure-function relationship in neuronal networks at different temporal and spatial scales. The latter range from fast-spiking and bursting dynamics of individual neurons organized in recurrent networks (Berner and Yanchuk; Protachevicz et al.; Rongala et al.; Sawicki and Schöll; Sánchez-Claros at al.), to neuronal populations' activity examined in terms of neural mass or neural field models (Al-Darabsah et al.; Bi et al.; Hutt et al.; Laing et al.; Tavakoli and Longtin) and to slow and ultra-slow fluctuations of neuronal and metabolic activity at the whole-brain scale (Coronel-Oliveros et al.; Gerster et al.).

Special attention is paid to the modeling of neuronal plasticity (Berner and Yanchuk), to the impact of time delays in coupling and intrinsic activity (Protachevicz et al.; Rongala et al., Sánchez-Claros at al.; Tavakoli and Longtin), and to the effects of noise or stochastic perturbations (Rongala et al.; Sánchez-Claros at al.; Tavakoli and Longtin), as well as to heterogeneity of individual and collective neuronal dynamics (Berner and Yanchuk; Bi et al.; Coronel-Oliveros at al.; Gerster et al.; Laing et al.; Tavakoli and Longtin; Zhou et al.).

A brief description of the contributions is reported below.

The experimental work of Zhou et al. investigates the properties of the local field potential (LFP) in the hippocampus and its spectra as energy is quenched from the system. The authors examine rat LFPs recorded from the hippocampus and entorhinal cortex during barbiturate overdose euthanasia. The data obtained in this study support the energy cascade theory where the energy flows from large cortical populations to smaller loops.

All other contributions report numerical or theoretical studies based on mean-field or network descriptions of neural systems. In particular, the following papers deal with neural mass and field models.

Laing et al. present a powerful method for studying the influence of a network structure on its dynamics by employing the reduction technique by Ott and Antonsen (2008). In particular, the authors investigate large heterogeneous networks of Winfree oscillators with various correlations in (meta-) parameters, such as degree or parameter assortativity.

Gerster et al. exploit the predictive power of personalized brain network models. The authors build multi-population neural mass models for a cohort of 20 healthy subjects and 15 epileptic patients, implementing next generation neural masses (Montbrió et al., 2015; Taher et al., 2020) for each brain region. As paradigms for testing the spatio-temporal organization, the authors systematically simulate the individual seizure-like propagation patterns.

Al-Darabsah et al. investigate the impacts of delays by modeling large interacting neural populations as neural-field systems. Using a master stability function analysis and numerical simulations, they find that delays can (1) stabilize brain dynamics by temporarily preventing the onset to oscillatory and pathologically synchronized dynamics and (2) enhance or weaken synchronization depending on the underlying eigenvalue spectrum of the connectivity matrix.

Bi et al. show that the E-I balance can cause various regimes observable in the brain. The authors classify the possible dynamical behaviors emerging in balanced E-I networks with structural heterogeneity. Analytic results show that both supra- and sub-threshold balanced asynchronous regimes are observable in the limit of large in-degrees. The coherent rhythms observed in the system can range from periodic and quasi-periodic collective oscillations to coherent chaos. These rhythms are characterized by regular or irregular temporal fluctuations joined to spatial coherence, similar to coherent fluctuations observed in the cortex over multiple spatial scales.

Hutt et al. derive a closed-form mean-field representation for an Erdös-Rényi network with two populations of interconnected neurons driven by additive noise. Considering Gaussian and Poissonian stimulation to excitatory neurons, they observe coherence resonance and show that partial stochastic stimulation promotes coherence resonance compared to global stimulation.

Coronel-Oliveros et al. consider a whole-brain model based on the Jasen and Rit neural mass Jansen and Rit (1995) and a human structural connectivity matrix, to find out which structural features of the human connectome network define the optimal neuromodulatory effects. They simulate the effect of the noradrenergic system as changes in filter gain, and studied its effects related to the global-, local-, and meso-scale features of the connectome.

Tavakoli and Longtin explore conditions under which additional delays in high-dimensional chaotic neural networks lead to a reduction in dynamic complexity, a phenomenon recently described as multi-delay complexity collapse. In particular, they observe that a global delayed inhibitory feedback can induce such a collapse.

The following contributions deal with recurrent networks based on spiking neurons or phase oscillators.

Protachevicz et al. study the effect of autapses by examining a random network with adaptive exponential integrate-and-fire neurons. They found that autapses can influence synchronous behavior in neural networks with excitatory synapses by either increasing or decreasing synchrony, depending on the parameters. However, when only inhibitory synapses are considered, synchronization does not suffer significant changes.

Rongala et al. explore noise and stability issues arising in recurrent neuronal networks. Their findings show that neuronal dynamic leak protects recurrent neuronal circuits from self-induction of spurious high-frequency signals. The authors test a range of models, from a linear non-spiking summation model to fully connected recurrent networks of excitatory and inhibitory neurons with randomly distributed weights and random sensory inputs.

Sawicki and Schöll discuss a minimal model that explains the modalities of the influence of music on the human brain. They report synchronization patterns induced by the sound frequency in a network of FitzHugh-Nagumo oscillators with empirically measured structural connectivity. The sound stimulus is modeled by an input to brain areas related to the auditory cortex. It is shown that the synchrony can be increased by properly adjusting the frequency and amplitude of the sound.

Sánchez-Claros et al. study the information flows in a canonical motif that mimics a cortico-thalamo-cortical circuit with three neuronal populations (V-motif). Through numerical simulations, the authors determine how the amount of information transferred between the populations depends on the connection delays and frequency detuning. The results highlight the role of the transthalamic V-motif in binding spatially separated cortical computations and suggest an important regulatory role of the direct cortico-cortical connection.

Berner and Yanchuk introduce a methodology for studying synchronization in adaptive networks with heterogeneous adaptation rules. The authors consider a network of phase oscillators with distance-dependent adaptations. For such system, the master stability function approach (Berner et al., 2021) is extended to networks with heterogeneous adaptation. Utilizing the proposed methodology, they explain mechanisms leading to synchronization or desynchronization by enhanced long-range connections.

The presented collection of papers in this Research Topic is united by the common theme of how the structure-function relationship contributes to our better understanding of this complex issue and can inspire further investigations in this direction.

## Author Contributions

All authors contributed to the article and approved the submitted version.

## Funding

This work was funded through the European Union's Horizon 2020 Framework Programme for Research and Innovation under the Specific Grant Agreement No. 945539 (Human Brain Project SGA3) and No. 826421 (VirtualBrainCloud). SY was supported by the German Research Foundation DFG, Project No. 411803875. JC was funded by the Portuguese Foundation for Science and Technology grants UIDB/50026/2020, UIDP/50026/2020, and CEECIND/03325/2017.

## Conflict of Interest

The authors declare that the research was conducted in the absence of any commercial or financial relationships that could be construed as a potential conflict of interest.

## Publisher's Note

All claims expressed in this article are solely those of the authors and do not necessarily represent those of their affiliated organizations, or those of the publisher, the editors and the reviewers. Any product that may be evaluated in this article, or claim that may be made by its manufacturer, is not guaranteed or endorsed by the publisher.
